# Real-time face keypoint detection for pre-anesthetic assessment with optimized YOLO11 model based on DeBiFormer

**DOI:** 10.3389/fmedt.2026.1780084

**Published:** 2026-05-13

**Authors:** Daotong Wang, Jianbo Su

**Affiliations:** School of Automation and Intelligent Sensing, Shanghai Jiao Tong University, Shanghai, China

**Keywords:** deep learning, keypoint detection, pose estimation, pre-anesthetic assessment, YOLO11-Pose

## Abstract

**Introduction:**

Pre-anesthetic assessment involves a series of evaluations for airway management. However, the lack of quantitative analysis may limit its reliability. In this study, we propose an optimized YOLO11-Pose framework with a DeBiFormer module for automated, image-based pre-anesthetic assessment.

**Methods:**

The proposed model performs single-stage inference to localize clinically relevant facial and hand keypoints associated with mouth opening, thyromental distance, and neck mobility. The framework was evaluated on a dataset collected from Ruijin Hospital under controlled acquisition conditions.

**Results:**

Experimental results demonstrated high detection performance. Measurement-based evaluations, including Bland-Altman analysis, pixel-level error, and normalized mean error (NME), indicated reliable agreement with reference annotations. In addition, Mallampati classification was evaluated to assess clinical applicability. The model achieved an accuracy of 77.34% in the four-class setting and improved performance in a clinically motivated binary setting, with an accuracy of 83.99% and a quadratic weighted kappa of 0.65, indicating substantial agreement.

**Discussion:**

These results suggest that the proposed method provides robust and clinically meaningful assessment of airway characteristics and may support automated pre-anesthetic evaluation in clinical practice.

## 1 Introduction

Difficult airways represent a critical challenge in anesthesia and are associated with severe complications and mortality when improperly managed (1). Despite the widespread adoption of video laryngoscopes, airway evaluation remains a fundamental clinical skill requiring accurate assessment and experience (2). Among various assessment strategies, the LEMON method provides a simple yet effective framework for evaluating airway risk, encompassing visual inspection, mouth opening, modified Mallampati classification, obstruction detection, and neck mobility (3, 4). Previous studies have demonstrated its high sensitivity in clinical practice (5–7). However, these measurements are largely subjective, leading to variability and inconsistency in clinical decision-making.

**Figure 1:**
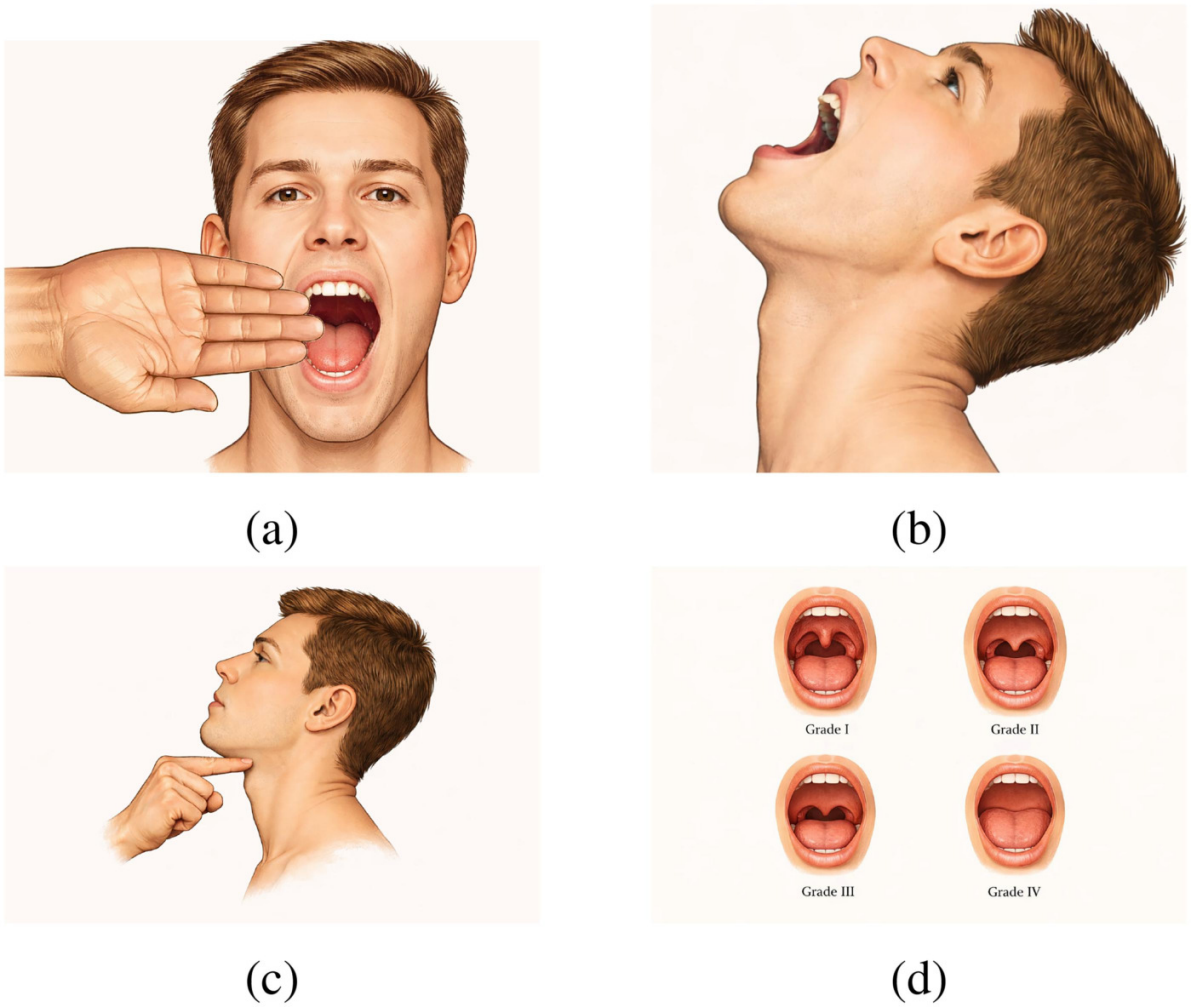
Diagram of difficult airway assessment. **(a)** Mouth opening; **(b)** Neck mobility; **(c)** Thyromental distance; **(d)** Mallampati.

In this study, we focus on the first three components shown in Figure 1, which are primarily associated with anatomical keypoint extraction.

To address the limitations of manual assessment, we propose a DeBiFormer-enhanced YOLO11 framework for craniofacial landmark detection (8, 9). The YOLO (You Only Look Once) series (10) is widely recognized for its real-time performance and competitive accuracy, and YOLO11 (11) further improves this balance. However, craniofacial landmark detection remains challenging due to the need to accurately localize small, low-contrast keypoints with high spatial precision. Standard architectures may lack the attention mechanisms required to capture subtle anatomical variations, especially under occlusion or pathological conditions (12–14).

To overcome these challenges, we integrate an optimized DeBiFormer module into the YOLO11 architecture, enabling adaptive feature refinement and improved focus on clinically relevant regions. This design enhances robustness and precision in keypoint localization while maintaining real-time performance. The proposed system is positioned as an intermediate module within a pre-anesthetic assessment workflow, focusing on automated keypoint extraction rather than direct clinical decision-making.

Furthermore, we evaluate the proposed method on a large-scale, clinically annotated dataset from Ruijin Hospital, providing a realistic benchmark for performance assessment and ensuring clinical relevance.

The remainder of this paper is organized as follows. Section 2 reviews related work, Section 3 presents the methodology, Section 4 reports the experimental results, and Section 5 concludes the paper.

## 2 Related works

Deep learning has significantly advanced image analysis, particularly in object detection and landmark localization, enabling automated extraction of clinically relevant anatomical features from medical images. The advent of sophisticated computer vision technology has gradually catalyzed a profound transformation in the field of medical image analysis. It offers a potential avenue for the automation of airway assessment. Early deep learning methods including two-stage landmark detection and direct regression methods have been widely used in medical imaging tasks requiring high localization accuracy (15). Nevertheless, these approaches often exhibit high computational complexity and are not always optimal for real-time clinical deployment.

One-stage detectors represented by the YOLO family have gradually emerged as mainstream solutions for scenarios requiring both speed and precision (16–18). YOLOv4 improved the balance between detection accuracy and efficiency, establishing a solid foundation for real-time object detection (16). Subsequent versions, such as YOLOv7 and YOLOv8, have further enhanced the representation ability of lightweight models by introducing improved feature pyramid fusion, decoupled heads and more effective loss functions (19, 20). In the domain of keypoint detection, YOLO-Pose extends the YOLO detection paradigm to human pose estimation by jointly regressing object bounding boxes and landmark positions, thereby enabling efficient multi-task optimization in a unified architecture (21, 22).

For craniofacial and medical landmark detection tasks, the challenge is not merely to identify large, salient structures, but to precisely localize small, low-contrast, and structurally correlated keypoints. Recent studies have demonstrated that high-resolution feature preservation and multi-scale context aggregation are critical for such tasks. Models such as HRNet maintain high-resolution representations throughout the network, thereby improving localization precision for anatomical landmarks (8). Concurrently, transformer-based architectures have demonstrated notable potential in capturing global contextual dependencies, which are frequently difficult for conventional convolutional backbones to model effectively.

In the context of airway or facial assessment, researchers have begun to explore the integration of attention mechanisms into detection pipelines (23–26). For example, attention-guided YOLO variants have been introduced to improve the discrimination of subtle anatomical features and to suppress redundant background information (20, 27). Nevertheless, conventional attention modules usually calculate dense interactions between all spatial tokens, which results in substantial computational overhead and insufficient flexibility in adapting to irregular anatomical structures.

In recent years, deformable attention has become a promising direction for enhancing the adaptability of visual models. Deformable convolution and deformable attention mechanisms enable models to focus sampling on informative spatial regions rather than relying on fixed receptive fields (12, 14). DeBiFormer advances this idea by introducing agent-based bi-level routing attention, which hierarchically selects relevant tokens and regions for feature interaction (13). This design is particularly appealing for landmark detection tasks in medical imagery, where local fine-grained details and global anatomical relationships must be modeled simultaneously.

Inspired by these developments, we propose a DeBiFormer-enhanced YOLO11 framework for automated pre-anesthetic assessment, aiming to improve the localization of clinically relevant keypoints while maintaining real-time performance.

## 3 Materials and methods

### 3.1 The general framework

The present work establishes a unified automated pre-anesthetic assessment framework based on YOLO11-Pose, specifically designed to evaluate key anatomical cues associated with difficult airway prediction. The overall architecture inherits the efficient single-stage paradigm of YOLO11 while incorporating a DeBiFormer-inspired deformable dual-level routing attention mechanism in the backbone to enhance fine-grained feature extraction and long-range dependency modeling.

The framework is designed to process three major visual assessment tasks involved in clinical airway evaluation: mouth opening, thyromental distance and neck mobility. These tasks require the accurate localization of multiple facial and cervical keypoints across heterogeneous image views. In contrast to conventional manual measurements, which rely on finger-width estimation or clinician experience, the proposed system formulates the problem as a unified keypoint-detection task and outputs anatomically meaningful coordinates directly from patient images.

The overall pipeline can be summarized as follows. First, the input RGB images are normalized and forwarded into the YOLO11 backbone. Second, an optimized backbone block enhanced with DeBiFormer is inserted to strengthen the representation of subtle local structures while preserving global anatomical context. Third, the neck and head layers adaptively process features corresponding to keypoint-relevant regions of interest at multiple scales. Finally, the decoupled prediction heads output the spatial positions and confidence values of the required facial landmarks.

The key design considerations of the framework include:

1. **Unified multi-task anatomical keypoint extraction.** A single network is designed to support different pre-anesthetic assessment components, thereby reducing the engineering complexity associated with maintaining independent pipelines for each measurement task.
2. **Deformable sparse attention for clinically relevant regions.** Instead of treating all image regions equally, the inserted DeBiFormer module dynamically focuses on semantically informative locations, such as the oral commissure, the mandibular contour and the thyroid cartilage region.
3. **Hierarchical contextual reasoning.** The dual-level routing mechanism facilitates structured interaction between local anatomical details and broader craniofacial geometry, which is crucial for stable keypoint localization under pose variation, occlusion and inter-patient variability.
4. **Compatibility with real-time deployment.** Despite the increased representational power, the overall design remains anchored in the efficiency advantages of YOLO11, enabling practical inference for bedside or workflow-oriented scenarios.

### 3.2 YOLO11-Pose architecture

YOLO11-Pose extends the classical YOLO object detection framework to multi-keypoint localization by integrating a pose estimation branch into the detection head. In the present study, the model receives patient facial or cervical images and predicts a set of pre-defined anatomical keypoints associated with different pre-anesthetic assessment tasks.

The architecture is composed of three principal components: the backbone, the neck and the head. The backbone extracts hierarchical visual representations from the input image, progressively encoding low-level edges, textures and landmarks into high-level semantic features. The neck aggregates multi-scale information and fuses features from different stages of the backbone to enhance the detection of targets with varying spatial sizes. The pose head then regresses both the object region and the coordinates of keypoints, thus supporting joint optimization of localization and landmark prediction.

In comparison with earlier YOLO-based pose models, YOLO11 introduces improved feature extraction and representation efficiency. However, for pre-anesthetic applications, the target landmarks are often small, weakly contrasted and influenced by variable patient poses. These properties place substantial demands on the network’s ability to preserve high-resolution anatomical cues while also modeling contextual dependencies between multiple landmarks.

To address this problem, the present work modifies the original YOLO11-Pose backbone by introducing an enhanced feature extraction module inspired by DeBiFormer. This modification is designed to improve the sensitivity of the backbone to key facial and cervical regions while maintaining the end-to-end inference capability of the original architecture.

### 3.3 DeBiFormer-enhanced backbone design

The proposed optimization targets the terminal backbone stage, where global semantic information is rich but fine anatomical detail may become diluted. The original backbone block is replaced or augmented with a DeBiFormer-inspired routing attention module to improve the representation of clinically critical landmarks.

The core motivation for this integration lies in the observation that pre-anesthetic assessment relies heavily on sparse but highly informative regions. Compared to conventional attention mechanisms, DeBiFormer provides more flexible spatial feature aggregation, which is particularly suitable for medical keypoint detection where anatomical structures may vary across patients. For instance, mouth-opening analysis focuses on the upper and lower lip center points together with the relationship to finger positions; thyromental distance estimation depends on the precise localization of the chin and thyroid cartilage; neck mobility analysis depends on the relative configuration of the eye and ear landmarks across extended and neutral positions. Conventional dense attention or uniform convolution may expend excessive computation on irrelevant regions while failing to allocate sufficient representational capacity to these subtle structures.

The DeBiFormer-inspired module addresses this challenge through deformable sparse feature sampling and hierarchical routing. At the lower level, deformable agent points learn to adaptively shift the sampling positions toward semantically meaningful regions. At the higher level, routed interactions enable the model to select which tokens or local regions should exchange information, thereby strengthening both local detail preservation and global anatomical consistency. The results in Table 1 are reported from the original publications under the COCO benchmark and are provided for qualitative comparison of different attention mechanisms. This comparison highlights the effectiveness of deformable routing attention in general vision tasks, which motivates its integration into our medical keypoint detection framework.

**Table 1:** Comparison of representative attention mechanisms on COCO dataset (results from original publications).

Method	mAP	AP50	AP75	APs	APl
Swin-T	41.5	62.1	44.2	25.1	55.5
DAT-T	42.8	64.4	45.2	28.0	57.8
RegionViT-S	43.9	65.5	47.3	28.5	58.0
BiFormer-S	45.9	66.9	49.4	30.2	61.7
**DeBiFormer-S**	**45.6**	**66.6**	**48.9**	**28.7**	**61.6**

Bold values indicate the best result among the compared methods in each column.

This mechanism can be conceptually summarized as follows. Let the query features be denoted by q and the reference point by p. The model learns an offset function to determine where feature sampling should occur, as defined in Equation 1:

$$\Delta p=\theta_{\text{offset}}(q).$$ (1)

The effective sampling position is then shifted from the original reference point to a more informative location, enabling adaptive attention to clinically salient structures. At the routing level, semantic affinity between grouped regions can be expressed as shown in Equation 2:

$$A^{r}=q^{r}(k^{r}{)}^{⊤},$$ (2)

where $q^{r}$ and $k^{r}$ denote region-level query and key embeddings, respectively. This regional interaction supports structured reasoning over related anatomical parts rather than isolated pixel-level responses.

**Figure 2:**
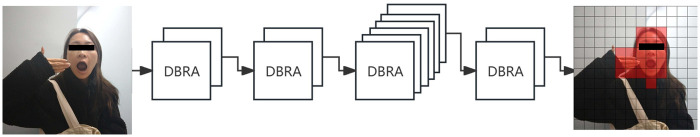
The architecture of DeBiFormer layer.

**Figure 3:**
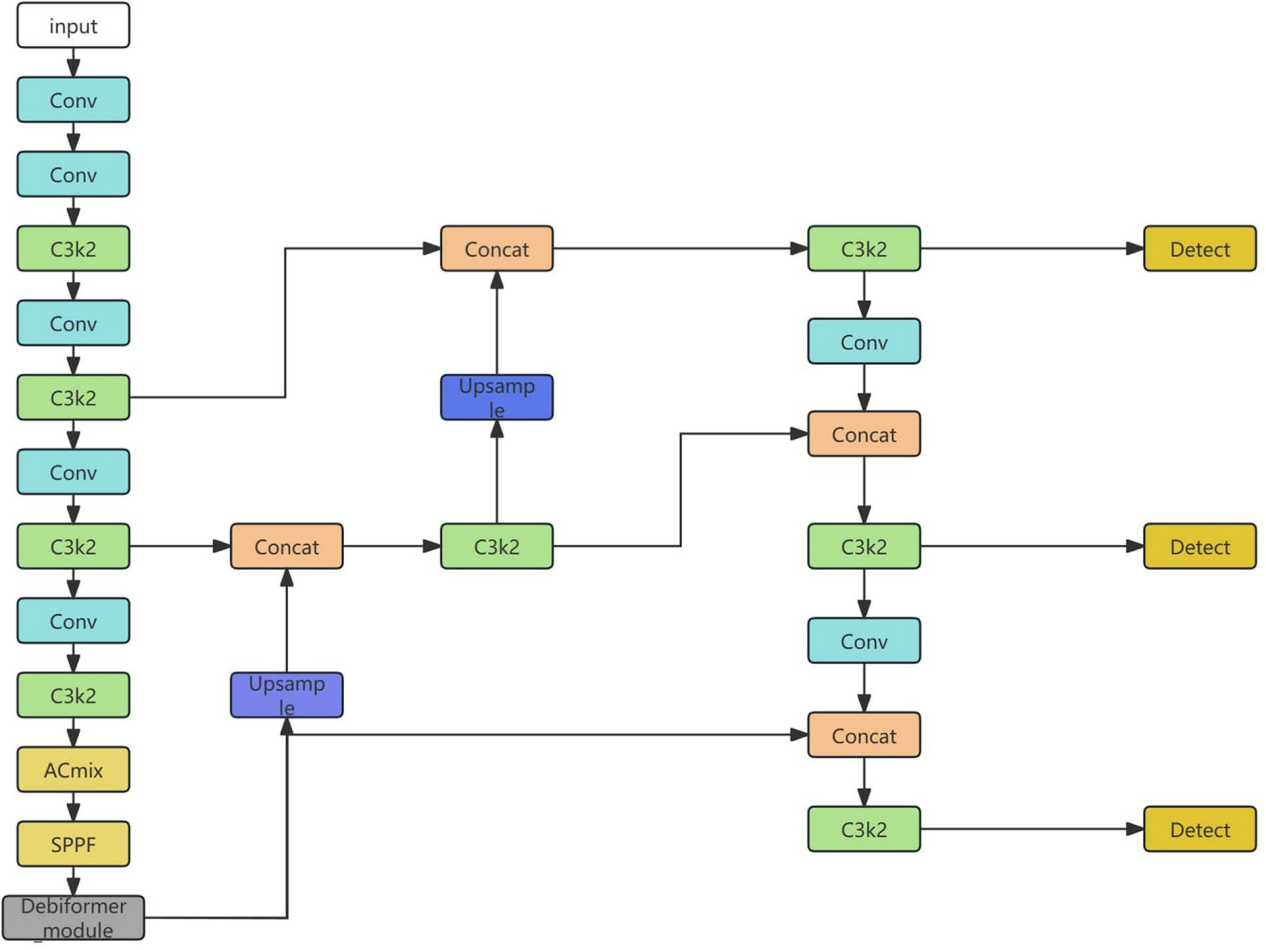
The architecture of modified YOLO11 network.

The proposed model incorporates the Deformable Bi-level Routing Attention (DBRA) module derived from the DeBiFormer architecture (Figure 2), which is designed to enhance adaptive feature aggregation through deformable routing mechanisms. In this study, the DBRA module is integrated into the YOLO11-Pose framework (Figure 3) to improve anatomical keypoint localization under low-contrast and noisy medical imaging conditions.

By integrating this module into YOLO11-Pose, the backbone gains the ability to model subtle local cues together with long-range dependencies among landmarks. This is expected to improve robustness under pose variation, partial occlusion and low-contrast imaging conditions while retaining the inference efficiency of a single-stage detector.

### 3.4 Clinical feature completeness in pre-anesthetic assessment

The concept of feature completeness in the context of the present work refers to the ability of the model to extract, preserve and integrate the multiple forms of anatomical information required for reliable pre-anesthetic evaluation. In contrast to standard keypoint tasks that focus only on positional accuracy, the proposed system is designed as an intermediate module to support downstream clinical measurements by ensuring that the extracted representation contains sufficient geometric, structural and contextual detail.

From a clinical perspective, the four tasks considered in this study involve distinct but complementary information. Mouth-opening evaluation requires both facial keypoints and hand-related spatial cues. Thyromental distance estimation relies on accurate identification of the chin and thyroid cartilage. Neck mobility assessment depends on stable relational modeling of the eye and ear landmarks under different head orientations. Mallampati classification segments 4 parts in oral cavity. Therefore, the network must not only detect points accurately, but also encode the anatomical relationships that permit clinically interpretable measurement.

In addition to keypoint localization and quantitative measurements, Mallampati classification was evaluated to assess clinically relevant airway grading. The original four-class Mallampati classification (Classes I–IV) was used as the primary task. Quadratic weighted Cohen’s kappa was computed to evaluate agreement between predicted and reference Mallampati classification, taking into account the ordinal nature of the classification. And in addition to the original four-class Mallampati classification (Classes I–IV), a clinically motivated binary classification was also evaluated, grouping Class I as normal airway and Classes II–IV as potentially difficult airway. This grouping reflects common clinical practice, where the primary concern is distinguishing difficult airway cases.

The proposed DeBiFormer-enhanced YOLO11 framework contributes to feature completeness in two aspects:

1. **Local detail preservation.** Deformable sparse attention enables the model to focus on subtle structures whose appearance is often easily diluted in conventional downsampling pipelines.
2. **Global structural consistency.** Hierarchical routing strengthens interactions between anatomically correlated regions, allowing the model to maintain coherent spatial predictions across the full craniofacial layout.

In this sense, the present method aims to move beyond pointwise detection toward a clinically meaningful representation of airway-related morphology. Such a representation provides a stronger basis for automated measurement, risk stratification and future multi-modal integration with other assessment signals.

## 4 Results

### 4.1 Dataset

The current database contains 4,556 images of 3,051 patients(including 1,091 images of mouth open, 1,073 images of thyromental distance, 1,960 images of neck mobility and 432 images of Mallampati). These images include mouth opening, neck mobility, thyromental distance and Mallampati. The data was collected using a 1080p RGB camera and dental endoscopy equipment. Automated modifications were implemented for the pre-anesthetic assessment process. Photographs of mouth opening, neck mobility and thyromental distance were taken with the patient seated 0.5 meters from the nurse. Photographs of mouth opening were taken with the patient facing the camera directly and holding up three fingers at the same level as their mouth with their right hand. Photographs of neck mobility were taken by rotating the patient through 90 degrees from the normal seated position to capture a side view. The thyromental distance was measured with the patient in the neck mobility position, with their head tilted back as far as possible. The patient extended the index finger of either hand, clenched into a fist, towards the thyroid cartilage. The entire database was divided into training, validation and test sets in a ratio of 7:2:1. Each patient corresponds to a single image in the dataset. Therefore, the random split inherently ensures that no subject appears in multiple subsets and no repeated acquisitions or multiple views were included for the same patient.

**Figure 4:**
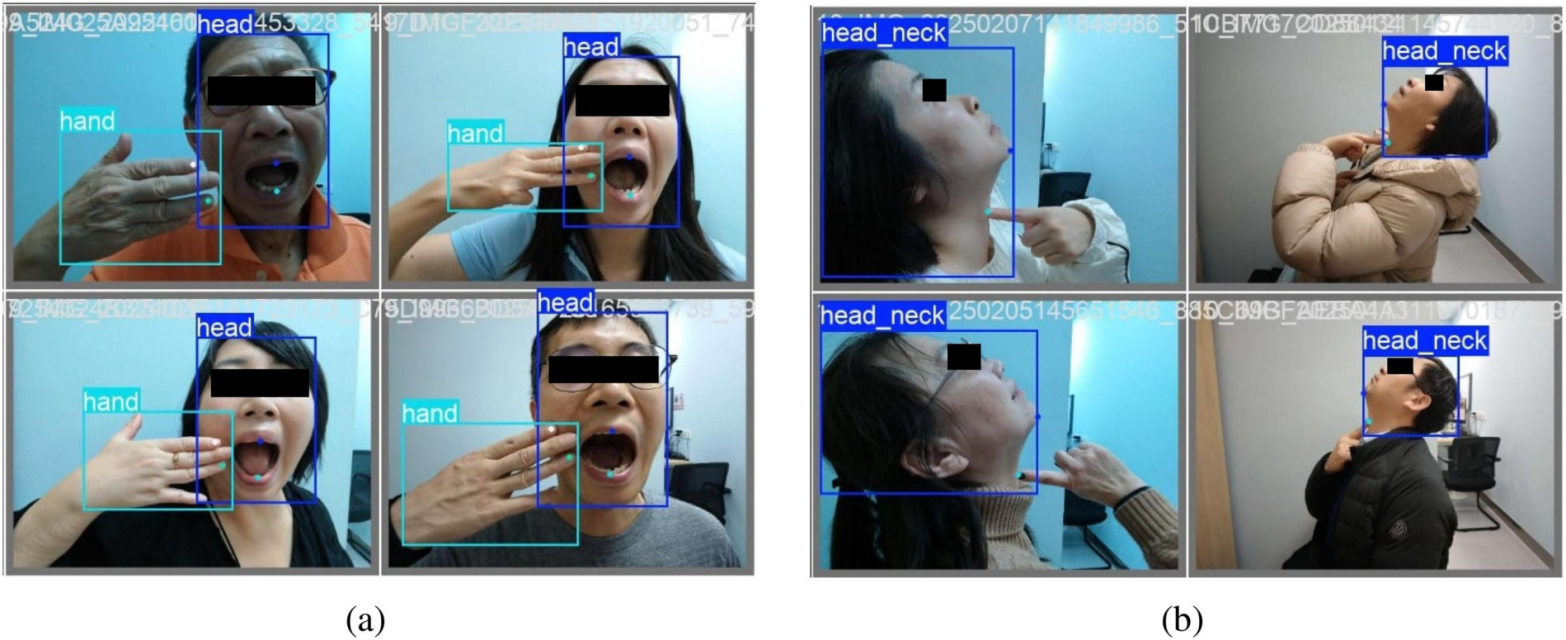
Examples of clinical annotation for airway assessment (mouth open & thyromental distance). **(a)** Annotations of mouth open, **(b)** Annotations of thyromental distance.

**Figure 5:**
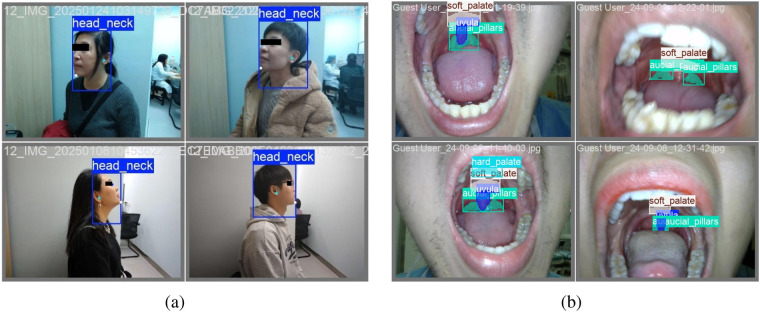
Examples of clinical annotation for airway assessment (neck mobility & modified Mallampati). **(a)** Annotations of neck mobility, **(b)** Annotations of modified Mallampati.

This pre-anesthetic assessment dataset was annotated by experts shown in Figures 4, 5. Key points on the face and oral cavity were annotated, and at least one image was collected per patient for each algorithm. All data underwent de-identification processing. The four algorithms are inter incisor gap, neck mobility, thyromental distance and modified Mallampati. The dataset is used to assess patient suitability for endotracheal intubation.

### 4.2 Pre-process

The database collection for this study was conducted in real clinical settings and was subject to various disturbances. For example, patient movement during image capture caused blurring and defocusing, introducing different types of noise and artifacts. To address this issue, the study employed simple data filtering methods and collaborated with experts to exclude unsuitable image samples. Furthermore, common YOLO data augmentation techniques, including MixUp, were employed to minimize data distortion and improve the network’s generalization capability.

### 4.3 Implementation details

This paper compares the original YOLO algorithm with an improved version using the collected database.

Selected frontal images of subjects with their mouths open and their hands extended flat with their fingers adjacent. The center points of the upper and lower lips were chosen and the index and ring fingers were designated for positioning. The thyromental distance algorithm captured frontal and lateral images from patients’ thyroid cartilage and chin. The neck mobility algorithm recorded images of the head with the neck extended. As for Mallampati, four regions of the oral cavity are identified: the uvula, soft palate, hard palate and buccal pillars. The original keypoints were generated using MediaPipe (28), with manual adjustments made to enhance accuracy. The original YOLO network model and the improved version were then trained separately. The learning rate decay, learning rate drop cycle and learning rate parameter were set to 0.1, 20 and 0.01, respectively. A total of 200 training cycles were performed, with a batch size of 1,000 and the Adam optimizer. The same conditions were applied to collect images for thyromental distance and neck mobility.

The entire architecture was implemented using the Ruijin Hospital database, which was used to evaluate performance and assist physicians with assessment-making. The proposed system adopts a client–server deployment architecture, where images acquired from a handheld device are transmitted to a server for processing. Therefore, the computational load is handled on the server side rather than on edge devices. Under this setting, the average inference time is approximately 7±2 ms per image, indicating that the proposed method satisfies real-time requirements in the current application scenario.

### 4.4 Evaluation metrics

In this paper, Precision(P), Recall(R) and mean Average Precision (mAP) are used to measure model accuracy, using the hospital committee annotations as the ground truth. The corresponding expressions are given in Equation 3:

$$\left\{ \begin{array}{cc} P & =\frac{\text{TP}}{\text{TP}+\text{FP}},\\ R & =\frac{\text{TP}}{\text{TP}+\text{FN}},\\ \text{AP} & =\overset{i=k-1}{\underset{i=0}{\Sigma }}[R(i)-R(i+1)]*P(i),\\ \text{mAP} & =\frac{1}{n}\overset{\;j=n}{\underset{\;j=1}{\Sigma }}{\text{AP}}_{j} \end{array}\right.$$ (3)

where TP, TN, FN, and FP stand for true positive, true negative, false negative, and false positive, respectively. k stands for number of thresholds, n stands for number of classes.

### 4.5 Performance

The experience is mainly conducted based on different versions of YOLO. YOLOv7 is applied as a tool for assesment of falling behavior in Pranavan et al. (19). It achieved a satisfied performance in keypoint dectection. YOLOv8 is a classic network. The head section in comparison utilizes different-sized feature maps to obtain category and position information for objects of varying sizes. Significantly reduction of the parameter size and computational complexity is based on the application of Distributional Focal Loss (DFL) (17). YOLOv8n is used for comparision to the detection performance. The original YOLO11 model achieves 90% precision for the current task. It is evident from the results presented in both Tables 2 and 3 that both approaches attained satisfactory classification performance levels on the hospital dataset. The segmentation performance for Mallampati classification is summarized in Table 4. The reported performance primarily reflects ROI (bounding box) detection accuracy rather than keypoint localization accuracy. Due to the relatively structured and controlled imaging conditions, ROI detection becomes a comparatively simpler task, leading to high mAP values. Therefore, ROI detection metrics alone are insufficient to evaluate the clinical reliability of the system. In this study, we further assess keypoint localization and measurement accuracy using pixel-level error, normalized mean error (NME), and Bland–Altman analysis.

**Table 2:** ROI detection performance of YOLO11-Pose under controlled experimental conditions.

Method	P	R	mAP	Parameters (M)	GFLOPS
YOLO11(mouth open)	99.5%	100%	98.7%	20.8	71.4
YOLO11(thyromental distance)	98.97%	100%	99.45%	20.8	71.4
YOLO11(neck mobility)	99.9%	100%	95.7%	20.8	71.4
YOLOv7	91.2%	100%	94.9%	80.2	101.8
YOLOv8	97.8%	100%	95.7%	3.4	9.7

**Table 3:** ROI detection performance of customed YOLO11-Pose under controlled experimental conditions.

Method	P	R	mAP	Parameters (M)	GFLOPS
YOLO11(mouth open)	99.5%	100%	98.8%	24.6	176
YOLO11(thyromental distance)	98.96%	100%	99.45%	24.6	176
YOLO11(neck mobility)	99.9%	100%	99.5%	24.6	176

**Table 4:** Segmentation performance of customed YOLO11-Pose under controlled experimental conditions.

Method	P	R	mAP	Parameters (M)	GFLOPS
YOLO11(Mallampati classification)	77.7%	78.2%	45.9%	24.6	176

In addition to detection metrics, we further evaluated the agreement between model predictions and clinician annotations for key clinical measurements in Table 5. Specifically, for mouth opening, thyromental distance, and neck mobility, the model achieved accuracies of 97.16% (1508/1552), 99.27% (1221/1230), and 83.54% (1406/1456), respectively. These results indicate a high level of consistency with clinician assessments in practical scenarios.

**Table 5:** Agreement between model predictions and clinician annotations for key clinical measurements.

Measurement	Correct/Total	Accuracy (%)
Mouth opening	1,508/1,552	97.16
Thyromental distance	1,221/1,230	99.27
Neck mobility	1,406/1,456	96.56

Our model provides a comprehensive evaluation with high precision of 99.17% on average and mAP at 97.1% on average within a unified framework. And the average inference time of approximately 7 ms per image under the server-based deployment setting, supporting its applicability in real-time clinical workflows. The model achieves precise integrated measurement of multi-scale, multi-modal physiological parameters. At the same time, it also enables automated, objective measurement of mouth opening and thyromental distance by precisely locating key points such as the corners of the mouth, the midpoint of the chin and the thyroid cartilage. This reduces subjectivity for traditional inaccuracy and improves consistency. This system not only automates manual measurement steps but also advances toward true automated risk assessment through deep learning models’ profound understanding of anatomical relationships. The rich key points and high-dimensional features extracted by the model lay a solid foundation for developing more complex predictive models in the future, ultimately achieving the leap from descriptive assessment to potential assessment support.

### 4.6 Measurement accuracy and agreement analysis

To further evaluate the clinical reliability of the proposed method, we conducted quantitative measurement analysis based on pixel-level localization error, normalized mean error (NME), and Bland–Altman agreement analysis.

Quantitative evaluation of measurement accuracy was conducted using pixel-level localization error and normalized mean error (NME), as summarized in Table 6.

**Table 6:** Measurement accuracy of the proposed method.

Task	Pixel error (px)	NME
Thyromental distance	20.148±0.732	0.027±0.001
Mouth opening	86.981±6.510	0.178±0.040
Neck mobility	13.084±0.540	0.026±0.001

**Figure 6:**
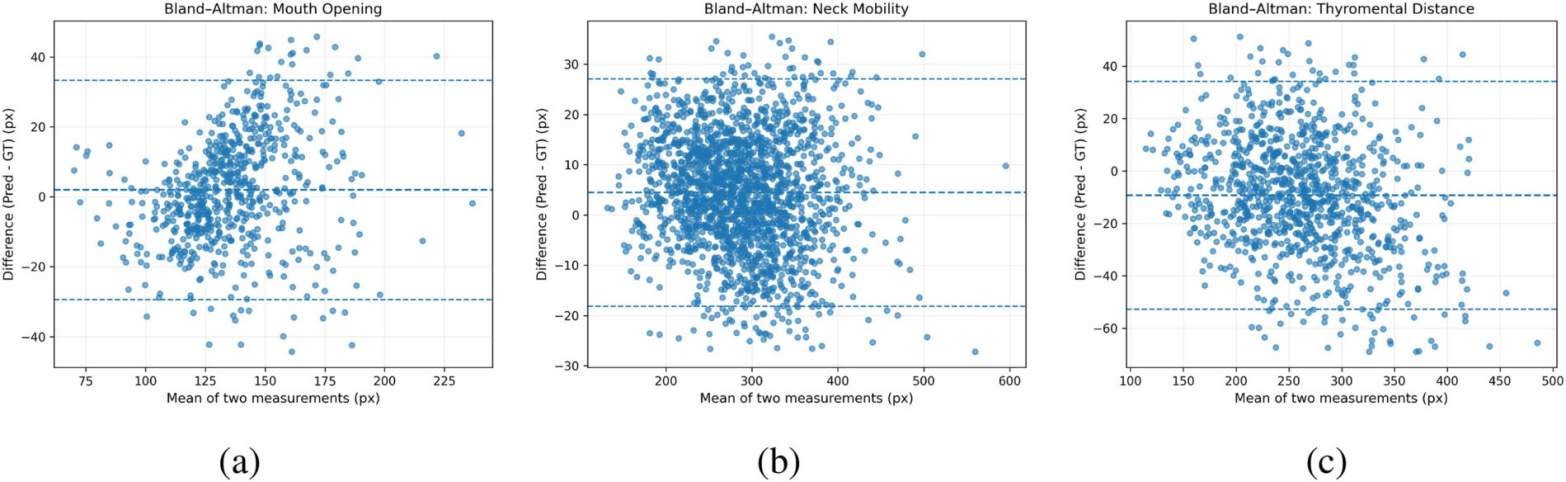
Bland–Altman analysis for keypoint localization. **(a)** Bland–Altman analysis of mouth opening, **(b)** Bland–Altman analysis of neck mobility, **(c)** Bland–Altman analysis of thyromental distance.

The Bland–Altman analysis was performed for three clinically relevant measurements, including mouth opening (lip distance), neck mobility (eye–ear distance), and thyromental distance, as shown in Figure 6. Outliers were excluded using the interquartile range (IQR) method to ensure robust statistical estimation.

For mouth opening (lip distance), the mean difference between model predictions and ground truth was 1.95 pixels, with limits of agreement ranging from −29.42 to 33.32 pixels. The relatively small mean bias indicates that the model does not exhibit systematic overestimation or underestimation in lip distance measurement.

For neck mobility (eye–ear distance), the mean difference was 4.50 pixels, with limits of agreement between -18.12 and 27.13 pixels. The distribution of differences was tightly concentrated around zero, demonstrating strong agreement and stable performance across a wide range of head poses.

For thyromental distance (chin-thyroid distance), the mean difference was −9.28 pixels, with limits of agreement from −52.72 to 34.17 pixels. Although a slightly larger variance was observed compared to the other measurements likely due to anatomical variation, the overall distribution remained centered with no significant systematic bias.

For Mallampati, the proposed method on Mallampati classification was evaluated. The original four-class problem (Classes I–IV) was additionally reformulated into a clinically relevant binary task: normal airway (Class I) vs. potentially difficult airway (Classes II–IV). The binary classification results were derived from the same model predictions without retraining.

While the four-class setting reflects the complete grading task, the binary setting provides a more clinically relevant evaluation.

For the original four-class Mallampati classification task, the model achieved an accuracy of 77.34% with a quadratic weighted kappa of 0.3591, indicating fair agreement. Most misclassifications occurred between adjacent grades, reflecting the inherent ambiguity of intermediate classes.

Under the clinically motivated binary setting (normal vs. difficult airway), the model achieved an accuracy of 83.99% and a binary weighted kappa of 0.65, indicating substantial agreement.

**Figure 7:**
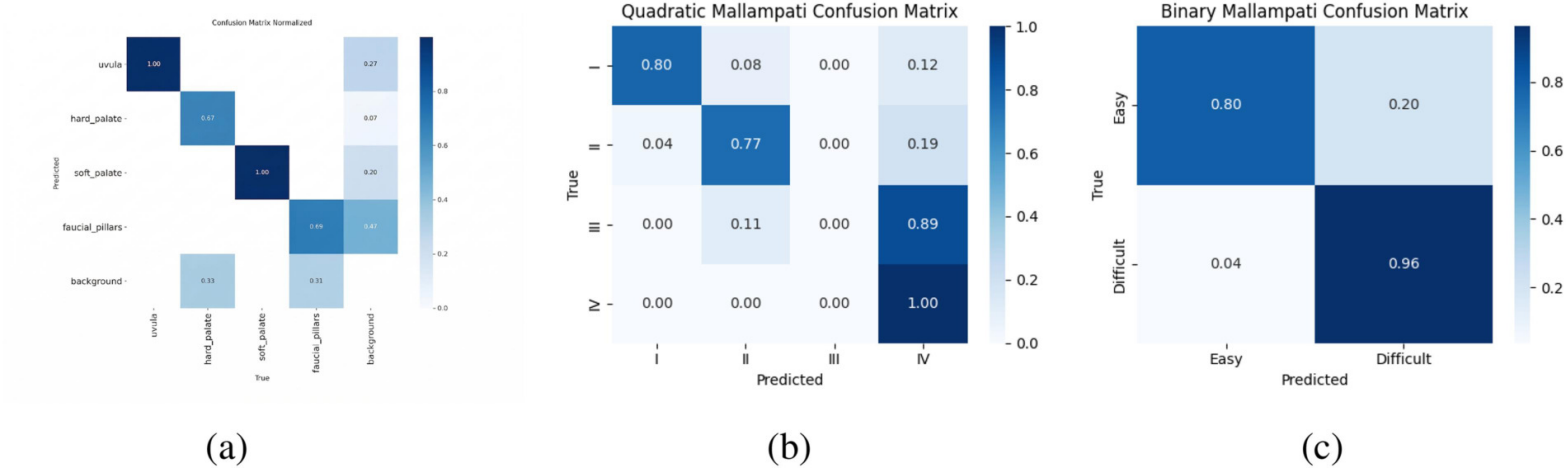
Results of Mallampati. **(a)** Normalized confusion matrix of 4 parts, **(b)** Normalized confusion matrix of quadratic classification, **(c)** normalized confusion matrix of binary classification.

The normalized confusion matrix (Figure 7c) shows that difficult airway cases were correctly identified with a sensitivity of 96%, while normal airway cases achieved an accuracy of 80%.

These results demonstrate that the proposed model is effective in identifying clinically critical difficult airway conditions.

Across all tasks, the results indicate that the proposed method achieves consistent and reliable measurement performance, supporting its applicability in automated pre-anesthetic assessment scenarios.

## 5 Discussion

In this study, we propose a DeBiFormer-enhanced YOLO11-Pose framework for automated keypoint extraction in pre-anesthetic airway assessment. The primary contribution of this work lies in improving the robustness and precision of anatomical keypoint localization under clinically relevant imaging conditions.

From a technical perspective, the proposed method introduces a deformable sparse attention mechanism that adaptively focuses on anatomically informative regions. This design allows the model to better capture subtle, low-contrast landmarks while maintaining global structural consistency. This is particularly important in medical imaging scenarios, where small spatial deviations may lead to clinically significant measurement differences.

Furthermore, the hierarchical routing mechanism enables structured interactions between anatomically correlated regions, improving prediction stability under pose variation, occlusion, and inter-patient variability. These properties make the proposed framework suitable as a reliable component for automated measurement in pre-anesthetic evaluation workflows.

Second, the integration of the DeBiFormer module increases computational complexity. Although real-time performance is achieved in the current server-based deployment setting (approximately 7±2 ms per image), future work will explore model compression techniques such as pruning and quantization to enable efficient deployment on resource-constrained devices.

The high mAP values observed in this study mainly reflect the ease of ROI detection under controlled acquisition settings, rather than the full complexity of anatomical keypoint localization. Therefore, measurement-based evaluation provides a more meaningful assessment of clinical applicability. The combination of pixel-level error, normalized mean error, Bland–Altman analysis and confusion matrix provides a comprehensive evaluation of both accuracy and agreement. Compared to detection-based metrics alone, these results offer stronger evidence of the model’s clinical validity and measurement reliability.

For mouth opening, the near-zero mean difference suggests that the model is capable of accurately estimating lip separation without systematic deviation. The observed spread of differences increases with larger opening values, which may be attributed to increased anatomical deformation and partial occlusion under wide mouth opening conditions.

For neck mobility, the results show the most stable agreement among all tasks, with a narrow distribution of differences. This indicates that the eye–ear geometric relationship is robustly captured by the model, even under varying head orientations and imaging conditions.

For thyromental distance, although the variance is relatively larger, the absence of significant bias suggests that the model maintains consistent measurement behavior. The increased variability may be related to differences in neck posture and annotation uncertainty in challenging cases.

Mallampati classification demonstrated improved agreement under the binary setting, reflecting the hierarchical nature of airway visibility. While fine-grained multi-class differentiation remains challenging, the model achieved high sensitivity (96%) for detecting difficult airway cases, indicating reliable performance in clinically relevant risk identification.

Overall, these findings support that the proposed system is capable of approximating clinician-level measurements with a high degree of consistency. This strengthens its potential as an objective and automated tool for pre-anesthetic airway assessment. Compared to purely detection-based evaluation, the inclusion of measurement error analysis and agreement assessment provides stronger evidence for the clinical validity and transparency of the proposed method.

Finally, several directions for future work should be considered. First, to address the limitation of single-center data, future studies will incorporate multi-center datasets with more diverse patient populations to improve the generalizability of the proposed framework. The relatively high performance observed in this study may be partially attributed to the controlled data acquisition conditions and limited variability of the dataset. Future work will focus on multi-center validation to improve cross-domain robustness. Second, model compression techniques such as pruning and quantization will be explored to enable deployment on resource-constrained edge devices. And broader comparisons with non-YOLO state-of-the-art methods will be explored in future work. Finally, future work will aim to establish stronger links between extracted measurements and clinically validated outcomes, enabling more comprehensive evaluation in real-world clinical settings.

## 6 Conclusions

The proposed methodology involves the utilization of a keypoint extraction network based on an enhanced YOLO model, with the objective of enhancing the extraction of keypoint features for a range of human face poses in real hospital settings. The introduction of a deformable dual-level routing attention mechanism has enabled the construction of a keypoint detection framework that exhibits exceptional feature completeness. This framework is designed to comprehensively encode multi-scale visual features that are required for pre-anesthetic assessment. These features span a range of scales, from macroscopic distance measurements to microscopic structural recognition, and from explicit anatomical landmarks to implicit semantic associations. This enhanced feature may improve clinical evaluation performance, enabling automated systems to conduct more comprehensive LEMON assessments with greater accuracy and robustness. The provision of reliable technical support is instrumental in facilitating the early identification of difficult airways and ensuring the precision of anesthesia.

## Data Availability

The datasets generated and/or analyzed during the current study are not publicly available due to institutional restrictions and data protection requirements. Although the images used in this study were de-identified, the dataset is derived from clinical materials and therefore cannot be shared publicly outside the institution. Requests for access should be directed to Daotong Wang, dtwang@sjtu.edu.cn.
